# Government veterinarians' perceptions of routine biosecurity focused on dairy cattle farms in north-western and north-eastern Spain

**DOI:** 10.3389/fvets.2023.1043966

**Published:** 2023-02-08

**Authors:** Sebastián Moya, José Navea, Jordi Casal, Giovanna Ciaravino, Eduardo Yus, Francisco Javier Diéguez, Bibiana Benavides, Francisco Tirado, Alberto Allepuz

**Affiliations:** ^1^Host-Pathogen Interactions (IHAP) - National Research Institute for Agriculture, Food and Environment (INRAE) - École Nationale Vétérinaire de Toulouse (ENVT), Toulouse, France; ^2^Latin American Center for Rural Development (RIMISP), Santiago, Chile; ^3^Department of Animal Health and Anatomy, Universitat Autònoma de Barcelona (UAB), Barcelona, Spain; ^4^Department of Animal Pathology, Universidade de Santiago de Compostela (USC), Lugo, Spain; ^5^Department of Anatomy, Animal Production and Veterinary Clinical Sciences, Universidade de Santiago de Compostela (USC), Lugo, Spain; ^6^Department of Animal Health, Universidad de Nariño (UDENAR), Pasto, Colombia; ^7^Department of Social Psychology, Universitat Autònoma de Barcelona (UAB), Barcelona, Spain

**Keywords:** biosecurity, content analysis, dairy cattle farm, interview, government veterinarian, perception

## Abstract

The implementation of biosecurity measures in livestock production systems can be affected by the psychosocial factors of its stakeholders, which can be observed through their knowledge, attitudes and perceptions/practices. In Spain, there are no regulations *per se* to promote biosecurity. Of all stakeholders, farmers and veterinarians have been addressed in previous biosecurity studies, but not veterinarians belonging specifically to the government services. This study explores this particular group's perceptions of routine biosecurity in livestock production systems in north-western and north-eastern Spain, an understanding of which could help to improve the implementation of biosecurity measures on farms. Eleven interviews were conducted with veterinarians from different levels of the government services in Galicia and Catalonia, and were analyzed through content analysis. Dairy cattle farms were considered as the reference livestock production systems. The respondents stress the limited availability of staff and time resources for biosecurity. The advisory role of government veterinarians is not well recognized among farmers, who feel that their services prioritize their sanctioning role. In fact, government veterinarians consider that farmers only implement biosecurity measures to avoid being sanctioned, and not because they are aware of the importance of biosecurity. Meanwhile, the participants comment that biosecurity regulations should be flexible and need to consider the contexts of the farms where biosecurity measures are implemented. Finally, government veterinarians are willing to attend biosecurity meetings together with all farm stakeholders, at which the government services could be informed about biosecurity issues on farms. The person who could take on the biosecurity advisory role should be defined, along with further discussion of such matters as the responsibilities of each stakeholder. Government veterinary services need to be considered in studies of biosecurity operations in order to improve their implementation. It is therefore concluded that government veterinarians are seeking to balance their own institutional perspective with that of farmers and veterinarians in the routine implementation of biosecurity measures.

## 1. Introduction

In livestock production systems, biosecurity can be defined as “*a set of management and physical measures designed to reduce the risk of introduction, establishment and spread of animal diseases, infections or infestations to, from and within an animal population*” (1). Biosecurity can benefit animal health and, consequently, the performance of livestock production (2). The implementation of biosecurity measures by farm stakeholders, such as farmers and veterinarians, can be influenced by individual, collective, local and general psychosocial factors. Individual factors include age and gender, whereby older farmers are stricter about the entry of animals of unknown health status and women have a higher level of education (3); information sources, for which purpose farmers can turn to veterinarians, magazines and media, other professionals, and the government (4, 5); education and knowledge, whereby farmers and veterinarians with higher levels in this regard are more willing to promote biosecurity and to invest money and time in it (3, 6–9); and risk-benefit perception, whereby more perceptive farmers and veterinarians prevent animals from interacting with others that are at risk of infectious disease, and less perceptive veterinarians in this regard do not consider themselves a risk, and do not organize their visits in consideration of the risk of a farm having an infectious disease (10–12). Collective factors include communication dynamics, whereby poor communication between veterinarians and farmers can negatively affect biosecurity (13, 14); and interpersonal relationships, where a trusting relationship between veterinarians and farmers encourages collaboration to improve biosecurity (15–17). Local factors include the location, size and infrastructure of farms, whereby family farms (which are smaller and older) implement fewer biosecurity measures (18–21). General factors include economics, whereby farmers who do not see the short-term economic benefits of biosecurity measures do not implement them (3, 9, 18, 22, 23); and legislation and government actions, where the absence of legislation and abundance of government bureaucracy makes farmers reluctant to implement biosecurity (24, 25). It is therefore plain to see that there are psychosocial factors that can *positively* or *negatively* affect the implementation of biosecurity measures on farms, and which can be observed through the knowledge, attitudes and perceptions/practices of stakeholders.

The European Union (EU) Animal Health Law (Regulation 2016/429 on transmissible animal diseases, 26) stresses the importance of biosecurity, not only in case of outbreaks of exotic diseases but also in day-to-day routines. This legislation encourages the development and establishment of biosecurity plans that are flexible and adaptable to different types of animal production, mainly considering local factors. In this sense, the Member States are encouraged to promote more detailed biosecurity regulations. In Spain, there are no current regulations *per se* on the implementation of biosecurity measures in livestock production systems. However, a regulation on minimum biosecurity measures is expected in the near future (27), which will give more competencies in animal health to the veterinarians belonging to government veterinary services.

Previous studies have shown that veterinarians are the main source of information on biosecurity for farmers and can influence their decision-making in a *positive way*, but also in a *negative way*. This is especially true with regard to government veterinarians (6, 18, 28–31), who farmers often conceive as “bad policemen” (18, 32). Together with distrust of the government services (33, 34), this has led farmers to not view these veterinarians as a source of advice (10). It has been pointed out that government veterinarians are unaware of the realities and problems of farms, and acquire a mainly sanctioning role, while their advisory role is only secondary. This might explain why the government veterinarians' advice may not be fully adapted to farms and why some farmers may not take this advice into account in their biosecurity decision-making (32).

Pig and poultry farms tend to have a high level of biosecurity due to mandatory measures, while cattle farms tend to have a low level due to such measures being voluntary and hence poorly implemented. Improved biosecurity in cattle farming could help prevent the transmission of zoonotic diseases that are such a threat to public health (35). In making these improvements, all stakeholders should be considered, including not just farmers and veterinarians, but also government veterinarians, for example. However, the biosecurity studies that have been carried out to date do not include government veterinarians as crucial actors in biosecurity, as they have mainly focused on the farmers and/or veterinarians that most frequently work on farms (6, 10, 18, 28–34). Knowledge of the regulations concerning government veterinary services and the farmers' opinions about government veterinarians in relation to biosecurity is important for animal health interventions on farms. It is also crucial to know more about government veterinarians' own opinions on farm biosecurity and the psychosocial factors that might affect the implementation of biosecurity measures in order to improve the services that they offer. The aim of this study was to explore perceptions of the implementation of routine biosecurity measures on livestock farms by government veterinarians in the Autonomous Communities (AC) of Galicia (north-west) and Catalonia (north-east) in Spain. In particular, it sought their opinion about government biosecurity services and their sanctioning and advisory actions; and on the government services' knowledge of the reality and problems of routine biosecurity on farms. This study is one of the first to consider and involve government veterinary services.

Eleven remote interviews were conducted with government veterinarians, which focused on dairy cattle farms in Galicia and Catalonia. The main findings were grouped into constraints on biosecurity implementation on farms, roles of government veterinary services, biosecurity awareness, biosecurity training, knowledge about farms, biosecurity meetings on livestock production, and mandatory biosecurity measures on farms.

## 2. Materials and methods

### 2.1. Study area

Spain has a total of 17 AC, which are regional entities with their own institutions and representatives and certain legislative, executive and administrative competencies, including animal health competencies. Each AC is divided into provinces, and each province into counties. This study was carried out in the ACs of Galicia (north-west) and Catalonia (north-east), mainly because the different types of dairy cattle farms in each region were considered to offer a good comparative framework, for the former are mainly family farms and the latter are mainly industrial (18, 19, 32), and so the similarities and differences between the participants' responses can be highlighted.

### 2.2. Participant recruitment

In Spain there are three types of veterinarians who usually work on livestock farms. First, there are private veterinarians, who advise farmers in technical areas related to herd health management. Second, there are veterinarians employed by the Health Defense Associations (HDA), which support farm associations with disease control programs. HDAs are managed by farmers and receive financial subsidies from the government services. Third, there are veterinarians belonging to the government veterinary services, who make sure that farmers and veterinarians carry out certain mandatory practices. This study focuses on this third type of veterinarian, who work on four different levels that are linked to each of the territorial divisions of Spain, the national, autonomous community, provincial and county levels. The national and autonomous community levels take a political-administrative approach with legislative implications, while the operations at the provincial and county levels are sanctioning-advisory through direct contact with farmers in their public offices or on farm visits.

For the purposes of the study, government veterinarians were considered to be the key informants. This is partly due to their knowledge and experience (36, 37), but also enabled the researchers to collect quality data in a short amount of time (38). Key informants were identified through an initial exploration of all structural and organizational levels of the government services according to the territoriality of Spain to ensure that the key informants offered a good representation of all these different levels.

Government veterinarians with knowledge and experience of biosecurity on livestock farms and who were known to be willing to participate in the study were contacted and recruited through purposive sampling. The authors used their research networks to contact government veterinarians with whom they had worked on other projects or studies. JC and AA contacted government veterinarians at the national and autonomous community levels in Catalonia, while EY and FD contacted other potential respondents at the autonomous community, provincial and county levels in Galicia. However, veterinarians at the autonomous community level also helped the authors to contact veterinarians from the provincial and county government services. These potential respondents were presented with a fact sheet that informed them about the objectives and characteristics of the study, stating that the topic was biosecurity with specific reference to Spanish dairy cattle farms, and particularly those in Galicia and Catalonia.

Eleven government veterinarians finally issued their informed consent to participate in the study, representing both the higher (national and autonomous community) and lower (provincial and county) levels. There were two from the national level (labeled N1 and N2), two from the autonomous community level (labeled GA and CA), three from the provincial level (labeled GP1, CP1, and CP2), and four from the county level (labeled GC1, GC2, CC1, and CC2) in both Galicia and Catalonia.

### 2.3. Data collection

Data was collected during the COVID-19 pandemic. Europe had one of the highest rates of positive cases in the world from early 2020 until early 2021, and Spain established containment policies, with a significant restriction of mobility between and within its territories (39, 40). Hence the data was collected by means of remote interviews *via* an online conference program, as in other fields of study (41, 42), which also offered advantages in terms of less displacement in the field and more flexibility in participants' schedules (43).

The remote interviews were conducted and recorded between 19 March 2020 and 19 October 2020. They were conducted by SM, who was at the time a PhD candidate and had conducted similar studies on dairy cattle farms with farmers and veterinarians (18, 19, 32). The interviews involved only the participants and SM and lasted between 45 and 120 min. They were semi-structured, which allowed the participants to express their views through their knowledge and experiences without following an established order (44). Two pilot interviews were conducted with government veterinarians at the national level, which were included in the study. The thematic guide included general and specific questions (Table 1). The former was related to the levels of government veterinary services and their resources, priorities, actions, proposals, and constraints in terms of biosecurity; and the specific questions were related to the reality and problems of farms, mandatory and minimum regulations, sanctioning and advisory roles, and participatory meetings. The interviewees were also able to add any additional information that they could offer. The interviews were subsequently transcribed and analyzed in their entirety by SM.

**Table 1:** Thematic guide on perceptions of government veterinary services in relation to biosecurity measures.

*General questions* - What was your previous profession? What is your current position? How long have you been in this position? What are your routine tasks in relation to animal health? - What resources do the government services have available to implement biosecurity measures? What resources do you use or have you used? Why? How are these resources managed? - What biosecurity measures are currently a priority in your position? Why? - What is the current position of government services on improving the implementation of biosecurity measures? What position should it take? What actions are being (or should be) taken to achieve this improvement? How are these actions being (or should these actions be) applied? What proposals exist (or should exist) to achieve this improvement? How are these proposals being (or should these proposals be) applied? Why? - What constraints exist (or could exist) to improve the implementation of biosecurity measures? Why? How relevant is the regulatory framework to these constraints? How can (or could) biosecurity measures be improved within this framework?
*Specific questions* - In previous studies, farmers and veterinarians have pointed out that the government services do not know the reality on farms, as the government services promote biosecurity measures that are difficult to implement. What is your opinion on this lack of knowledge? Why? What is the reality, or the problems, on farms in relation to biosecurity? How could the reality and problems on farms be better known? - In previous studies, farmers and veterinarians have pointed out that biosecurity measures from the government services are perceived with reluctance, due to the few arguments to justify its implementation. What is your opinion on this situation? What is your position on the mandatory nature of some biosecurity measures? What biosecurity measures should be mandatory? What biosecurity measures should be minimum? How could these measures be implemented? What position should the government services take on these mandatory and minimum measures? Why? - In previous studies, farmers and veterinarians have pointed out that the government services have mainly a sanctioning role but also an advisory role. What is your position on the sanctioning role of the government services as perceived by farmers and veterinarians? What is your position on the advisory role that the government services should have with farmers? What actions should be taken to achieve this role? How? - Finally, farmers and veterinarians share the idea that there should be biosecurity meetings between all actors. What is your opinion on these meetings? Why? What constraints are there (or could there be) to holding these meetings? How could these meetings be held? What is your position on binding participatory processes?

### 2.4. Data analysis

The interviews were analyzed through content analysis using the qualitative ATLAS.ti software. The approach proposed by Elo and Kyngas (45) and Elo et al. (46) was considered. As biosecurity was being explored through a limited number of participants, a mainly deductive logic was used (47). The transcriptions were read for a general understanding of their content, and then established codes (i.e., deductive approach) were created along with their meaning units (Table 2), which are sections of text that are related to the objective of the study. Each meaning unit was coded according to its content in the established codes, but emerging codes (i.e., inductive approach) were also created as the texts were analyzed. Finally, all codes were compared, and similar codes were grouped and labeled into categories. Preliminary categories were discussed and agreed between SM and AA.

**Table 2:** Codes and meaning units in content analysis.

**Codes**	**Meaning units**
Measures	Biosecurity measures that are implemented on farms according to government veterinarians.
Constraints	Constraints to implement biosecurity measures according to government veterinarians.
Resources	Resources available to government veterinary services to lead the implementation of biosecurity measures according to government veterinarians.
Roles	Sanctioning and advisory role of government veterinary services and its approaches and consequences according to government veterinarians.
Importance	Importance of biosecurity measures for government veterinary services and farmers according to government veterinarians.
Awareness and training	Awareness and training programs for farmers, veterinarians, and government veterinarians according to government veterinarians.
Knowledge	Knowledge of the reality and problems of farms according to government veterinarians.
Meetings	Feasibility of holding meetings between different stakeholders and government veterinary services according to government veterinarians.
Mandatory	Mandatory biosecurity measures on farms according to government veterinarians.

For validity and reliability of findings, sampling adequacy, positionality, data triangulation, peer debriefing and methodological consistency were included (48, 49). Sampling adequacy was evidenced through data saturation (50), which is the point when participants' statements are merely repeated without providing new findings. Regarding positionality, the authors adopted a critical stance in relation to the participants' statements, which involves questioning the reasoning and making judgements based solely on these responses. Regarding data triangulation, previous studies on biosecurity on dairy cattle farms were used to compare the opinions of farmers and veterinarians with those of government veterinarians. For peer debriefing, meetings were held with all authors, specifically between SM and each of the authors separately. At these meetings the authors shared their ideas and interpretations with regard to the final categories. For the purposes of methodological consistency, congruencies were found between the research questions, the materials and methods used, and the research results.

Finally, it is important to note that only the codes with their respective quotations linked to the study objectives were incorporated in the results. Representative quotations were included for illustrative purposes. These quotations were selected by SM and AA and translated from Spanish into English. The quotations were presented in relation to the four existing levels of government veterinary services.

### 2.5. Ethics statement

The Ethics Committee of the Universitat Autònoma de Barcelona approved the study proposal (CEEAH 4055) and helped design the informed consent for participants, which explained the objective of the study and the conditions and guarantees of its participants. It also stated that the data collected would be confidential and anonymous, that there would be no financial benefit for participating, and that the interviews would be audio and/or text recorded. The decision to participate in the study was entirely voluntary, and participants could stop and leave the interview at any time they wanted. The informed consent was then signed by the participants and SM, with both parties receiving a copy.

## 3. Results

### 3.1. Constraints on biosecurity implementation on farms

The provincial and county levels pointed out that farmers' economic resources may be a constraint for the implementation of biosecurity measures, such as wheeled rains, footbaths, changing rooms, animal quarantine, animal unloading and loading areas, and perimeter fencing. However, the national levels commented that farmers sometimes used this as an excuse to skirt their responsibilities, for they also fail to implement measures that do not require a major financial investment, such as records of the entry and exit of people and vehicles, transit from clean to dirty areas, and non-shared tools in animal handling. Furthermore, government veterinary services were aware of other constraints on the implementation of biosecurity measures, such as small farms with small herds, which are usually family-run, and farms with an atomised infrastructure or which share the same roads with other farms. However, the autonomous community levels believed that if the sector were to request incentives for biosecurity, the government services could set up subsidization programs for this purpose.

All levels of government veterinary services agreed on the limited budgetary resources allocated to biosecurity, with the exception of those associated with disease eradication programs (e.g., cleaning and disinfection procedures in positive cases). However, the provincial levels noted the existence of budgets for biosecurity training programs, mainly through agricultural schools, while the autonomous community levels considered that the staff of government veterinary services working in biosecurity were also a resource. However, the autonomous community representatives pointed out that these staff are limited in number and the time available to perform these tasks due to overwork in other areas.

CC2: “(…) There is a limitation on staff to monitor more directly on a day-to-day basis (...). The only problem is that we cannot reach all the farms. If we could inspect all the farms every year, it would raise the quality of the sector (…)”

County levels mentioned that the existence of regional administrative divisions was a constraint. They felt that government veterinarians from different levels of the organizational structure could have different criteria for the implementation of some biosecurity measures and that this generates comparative grievances.

### 3.2. Roles of government veterinary services

Government veterinary services recognized that they could serve a sanctioning or advisory role, but that they should not be paternalistic toward farmers. The national levels did not agree with the sanctioning role either, as it left aside actions focused on ensuring public health in terms of preventing the entry and spread of infectious diseases. Furthermore, the autonomous community levels stressed that farmers should be made aware of the importance of biosecurity measures and not only implement them to avoid being sanctioned.

CA: “(…) I don't think that the government services should be seen as a sanctioning entity (...). If you implement biosecurity measures, your animals are healthy. However, biosecurity has to be here [inside your head], not in files (...). It is a change of thinking, not to avoid a fine (…)”

On the contrary, government veterinary services commented that sanctions allow biosecurity measures to be implemented. In spite of this, the county levels highlighted that the resources that farms possess should be considered before applying sanctions, as some farms are limited in this regard. Besides, the consideration of these resources could help to further adapt biosecurity measures on farms through enforceable legislation for farmers.

In relation to the advisory role toward farmers, the autonomous community levels pointed out that the government services should mainly enforce compliance with regulations, generally applying sanctions. Meanwhile, the provincial and county levels commented that whenever possible government veterinarians should try to inform and train farmers on legislative and practical aspects prior to the issue of a sanction, through courses or visits by farmers to their services, for instance.

The autonomous community levels recognized that the sanctioning role of government veterinarians can be a drawback as it means they tend to be perceived in a negative light. In contrast, the autonomous community representatives wish to be positively perceived for their advisory role. To address this drawback, the provincial levels suggested that it would be ideal to have two different teams, one of inspectors and the other of advisors, to fully develop these functions.

CP2: “(…) It is difficult to give advice when you carry out inspections (...). It would not be a bad idea to create a body of inspectors, one the person for inspection. If you get it wrong, you get it wrong (...). And then there should be other veterinarians [who would also be part of government veterinary services as inspectors] who, without being inspectors, can give advice to the farms (…)”

The provincial representatives criticized the fact that the government services have been prioritizing their sanctioning role over their advisory one, as this advice was previously offered by the agricultural extension services, which do not exist anymore.

### 3.3. Biosecurity awareness

The government veterinary services want all livestock farms to implement the different biosecurity measures adequately. However, according to its veterinarians, farmers implemented different measures in a heterogeneous manner. For example, at the provincial levels, measures related to animal movements were efficiently and effectively implemented, as the farmers were aware of their importance, but this was not the case with measures related to records of the entry and exit of visitors (both persons and vehicles) to and from farms. The provincial representatives believed that inadequate implementation of biosecurity measures was mainly due to traditions and acquired farm routines, which are difficult to change.

GP1: “(…) The sector tries to do things well here (...), but very often they have acquired biosecurity routines. It is difficult to change these routines because their parents and grandparents already implemented them (…)”

For county levels, farmers were not aware of the importance of biosecurity due to overwork in other areas, such as managerial duties. This was evidenced by the fact that farmers only implemented biosecurity measures to obtain subsidies.

On the other hand, government veterinary services also had different degrees of awareness of biosecurity measures, both between different levels and within the same level of the organizational structure of the government services. For instance, the county levels considered farm records of entry and exit of visitors to be important, as these records allow efficient traceability, and may establish comprehensive disinfection points or restrict entry to and exit from farms. Meanwhile, the autonomous community and other county levels considered animal movements to be crucial. However, the county representatives recognized that these movements were often not audited, as these biosecurity measures depended on the awareness of farmers. Despite the above, government veterinary services thought that new dairy cattle farms should comply with a set of minimum biosecurity measures, while old farms should also comply, but with certain deadlines; although for the provincial levels these biosecurity measures might already be established in the autonomous community legislation. Nevertheless, the government services believed that the implementation of minimum biosecurity measures should be flexible and consider the conditions of farmers and their farms.

CP2: “(…) A program with some minimums and then you adapt it. However, the minimums must be mandatory (...). Three or four minimum biosecurity measures if necessary, and then you adapt them to the conditions of the farmer and the farm (…)”

### 3.4. Biosecurity training

For the national levels, farmers did not recognize their own responsibility for implementing biosecurity measures due to a lack of training and, consequently, a lack of awareness of their importance. In this regard, the provincial levels stressed that there should also be mandatory training for all farmers to understand the rationale behind biosecurity implementation.

CP1: “(…) We try to provide training so that people try to understand what they are obliged to do (...). I believe that the function of the government services is to legislate and try to train people voluntarily or mandatorily so that they understand the regulations (…)”

Government veterinary services pointed out that farmers not only had a lack of training due to time restrictions, but also that farmers were reluctant and unwilling to understand the rationale of biosecurity. For example, farmers did not agree with some biosecurity measures, such as perimeter fencing, because of the feasibility issues. In contrast, the provincial levels commented that although government veterinarians constantly try to train farmers on infectious diseases, farmers were already aware of their consequences and, therefore, of the objectives of biosecurity measures.

On the other hand, government veterinarians commented that other training tools should be developed to make farmers aware of the importance of biosecurity. In this respect, the national levels highlighted the challenges associated with increased biosecurity awareness among farmers through training. These challenges were due to the absence of outbreaks of exotic diseases in the country for several years and veterinarians prioritizing fields other than prevention, such as nutrition and reproduction. In this regard, the national representatives mentioned that government veterinary services try to train veterinarians in animal health (including infectious diseases and biosecurity) to transmit a unified message to farmers. And in turn, the national levels believed that the government services should not only be responsible for the technical training of veterinarians, but also for raising awareness among sectorial associations, as the government services had limited resources for this.

N2: “(…) Veterinarians are a group in which we invest a lot of resources, perhaps a little more technical than those dedicated to awareness-raising (...). Awareness-raising in my opinion should be approached from the point of view of the sectorial associations (…)”

The national representatives commented that all government veterinary services were also trying to raise biosecurity awareness, not just among farmers, but also among veterinarians.

### 3.5. Knowledge about farms

Government veterinarians were aware of the reality and problems of farms. However, while the county representatives were constantly in contact with farms, they acknowledged that the higher levels might have inaccurate knowledge about them.

CC1: “(…) People indeed have the perspective that those who legislate [higher levels] do not know what the reality is. However, people who are at the lower level like me (...), I know the farmers perfectly well, I know not only their way of working but also their problems (…)”

For government veterinary services, the higher levels should have more contact with farms and their farmers and veterinarians. They also acknowledged that spending most of their time in the office was a constraint to understanding the problems on farms, and that the different levels of government veterinary services, together with their respective tasks, seems to influence and affect the flow of information about what really happens on farms.

N2: “(…) There are many levels of government veterinary services with many people, there are many realities (...). We try to be very aware of the reality of the field. The problem is that sometimes in the flow of information, the information does not arrive or arrives badly or incompletely (…)”

In this respect, the government veterinary services wished to hold meetings to learn more details about the reality and problems of farms, where there would be a reciprocal flow of information between both parties (the government services and farms).

### 3.6. Biosecurity meetings on livestock production

In relation to the organization of meetings on biosecurity with all stakeholders (farmers, representatives of farm associations, private veterinarians, veterinarians employed by the HDA and government veterinarians), the government veterinary services proposed voluntary attendance. In addition, while the national levels addressed the importance of biosecurity whenever possible in meetings on other topics, government veterinarians commented that time was the main constraint on attendance of biosecurity meetings. The autonomous community and provincial levels pointed out that these meetings could lead to consensual advice developed by all stakeholders to facilitate awareness-raising among them. In fact, for the government veterinary services, these meetings could allow farmers to learn from each other, but they should strengthen their social networks beforehand in order to present their collective demands to the government services.

GA: “(…) Farmers do not move anything either, they do not get together for anything. The logical thing would be for them to get together among themselves and to make an effort to solve the problems, or to raise these problems with the government services (...). This should be the starting point for farmers (…)”

On the contrary, the autonomous community levels sense the need to explore the scope of consensual advice in accordance with legislation. The government veterinary services pointed out that these meetings should be managed by the farmers' unions, which should propose initiatives on biosecurity, and not by the government services, who lacked the means to convene them.

Regarding the participation of farmers in biosecurity meetings, the autonomous community levels sense drawbacks, as farmers prioritize their political positions over possible solutions to their biosecurity problems. Similarly, for government veterinarians, another drawback was the time spent at biosecurity meetings, as farmers could get tired of them and not attend, even if they have a voice at them. Furthermore, the provincial levels pointed out that the viability of holding these meetings with farmers and veterinarians was low, as the two groups were worlds apart, with their own languages, understandings, and interests. The provincial representatives pointed out that there might be areas that should not be dealt with at these meetings, such as purely legislative issues. Despite the above, the provincial levels commented on the existence of sectoral round tables, with representation of the different groups in the sector, in which various issues, such as biosecurity, were discussed. Indeed, the national levels highlighted the so-called “local sanitation commissions” that still existed in some places, where representatives of the different groups in the sector meet to coordinate actions on an annual basis, although these commissions were disappearing due to the limited availability of staff.

N1: “(…) The legislation described the so-called “local sanitation commissions” (...). Before starting an action in a municipality, we called together all the farmers, the representatives of the municipality, and the veterinarians in the area. And the government services would explain what we were going to do (...) so that these people could tell us what problems they saw with that action (…)”

### 3.7. Mandatory biosecurity measures on farms

The county levels thought that mandatory biosecurity measures would allow all actors in the sector to implement them to higher biosecurity standards, which would also boost their public image.

CC2: “(…) There would be no problem with making biosecurity mandatory, with high standards, because farmers are also interested in having a good public image (...), a good image of good biosecurity (…)”

On the other hand, the national levels pointed out that considering biosecurity measures as an imposition is a mistake, as the sector should really be made aware of the need to achieve these standards. Besides, according to the national representatives, farmers conceived government veterinary services as an enemy because of the mandatory biosecurity measures that its veterinarians had to enforce. However, the government veterinary services also felt that this conception was changing because they were getting closer to the farmers. In addition, the national levels also commented that these measures had been permissive despite their mandatory nature.

## 4. Discussion

The results of the present study have presented the perceptions of government veterinarians of the implementation of routine biosecurity measures in Galicia and Catalonia. In particular, these results revealed their opinions of government veterinary services with regard to biosecurity and their sanctioning and advisory actions, and of their own knowledge of the realities and problems of routine biosecurity on farms.

Government veterinary services have limited resources for biosecurity, which tend to be focused on animal health programs, such as bovine tuberculosis (TB) (51) based on Royal Decree 2611/1996 (52), or infectious bovine rhinotracheitis (IBR) based on Royal Decree 554/2019 (53). However, the government veterinarians also mention the possibility that farmers could apply for subsidies for certain biosecurity measures. In fact, these subsidies could be beneficial as incentives for farmers to implement biosecurity measures and, in turn, make the government services aware of farmers' biosecurity needs (54, 55). However, they must be accompanied by awareness-raising to ensure that the routine implementation of biosecurity measures is efficient and effective.

Regarding the roles of government veterinary services, their veterinarians feel that the sanctioning and advisory roles perceived by farmers and veterinarians were equally necessary, even though the former is more recognized than the latter. One of the possible reasons for this recognition may be the limited resources available to the government services in terms of staff and time. This may also affect the training and advice that government veterinary services can offer to farmers, which is not viewed as efficient or effective in terms of their impact on farmers, a situation that could be evidenced by the advisory role being under-recognized (32).

The advisory role of government veterinary services was previously served by the agricultural extension services in the form of technical advice on efficient and effective practices, organization of training, refresher programs and technical-scientific dissemination events (56). These objectives could also be taken up by government veterinary services. However, there is an interest in reinforcing this advisory role toward farmers, possibly though the veterinarian responsible for each farm [Regulation 2016/429 (26); Royal Decree 993/2014 (57); Law 8/2003 (58)], or by a veterinarian belonging to a HDA. The latter also serve an advisory role in the routine implementation of biosecurity measures for TB and bovine viral diarrhea (BVD). In this respect, irrespective of who takes on this advisory role, this person could not only complement the government veterinary services, but also private veterinarians, who need to improve their biosecurity and infectious disease awareness (59–62).

Some government veterinary services highlighted a lack of biosecurity awareness among farmers, which could be the fault of government veterinarians, or of private veterinarians, to whom farmers often turn to for reliable information (63). Hence it could be interesting to evaluate the impact of the sanctioning and advisory roles served mainly by the government veterinary services, but also by private veterinarians, regarding the ultimate day-to-day implementation of biosecurity measures by farmers. Indeed, both roles could be rethought and new awareness strategies could be proposed. For example, in relation to the advisory role, the government veterinarians did not mention their own training in teaching skills, which some levels of the government veterinary services might not be receiving. This could in turn affect the training that government veterinarians provide to farmers. The government veterinary services should therefore not only offer training on biosecurity and infectious diseases, but also on teaching skills, at least for the county levels that have the most contact with farms. This training should not only consider routine biosecurity, but also outbreaks of exotic diseases, for which the sector is often unprepared.

The biosecurity training that farmers receive could also suffer from other drawbacks. For example, it might not consider the particular conditions of farmers and their farms (20), and different biosecurity materials might offer contradictory advice, leading farmers not to implement biosecurity measures out of confusion (64). Therefore, the training that farmers receive should consider their particular contexts, as well as provide unified advice based on scientific evidence, which the government veterinarians are aware of from their day-to-day routines.

According to the participants, certain biosecurity measures on dairy cattle farms are often not audited. We could note here that private veterinarians are not entitled to audit biosecurity measures in these production systems, but they can give advice and negotiate with farmers on biosecurity implementation. In contrast, veterinarians employed by HDAs can audit biosecurity measures linked to control programs for TB and BVD, although many farms are not members of a farmers' association and therefore have no contact with any HDA veterinarians. Hence, in order for biosecurity measures to be fully audited by these HDA veterinarians, all farms would need to join a farmers' association. Alternatively, HDA veterinarians could be a support for the government veterinary services when the legislation comes into force, which will endow them with greater competencies in terms of animal health.

The government veterinarians were aware that family farms face more constraints on the implementation of biosecurity measures than industrial farms. They did not offer details about the differential treatment of family farms, possibly to avoid causing grievances, but they did mention that older farms (which tend to be family farms) should have more time to implement minimum biosecurity measures than newer farms. It would be interesting to look further into the different treatment of family and industrial farms by government veterinarians in relation to particular biosecurity measures.

In previous studies on biosecurity on dairy cattle farms, farmers and veterinarians pointed out that the government services were unaware of the reality and problems on farms, as they generated biosecurity regulations and legislation that were complicated to implement on a day-to-day basis (18, 32). However, the government veterinary services agreed that only the higher levels, such as the national and autonomous community ones, were unaware of the reality and problems that farms face. To address this, the government veterinarians have agreed to hold meetings specifically on biosecurity that all actors in the sector could attend on a voluntary basis. However, although similar meetings already exist, they mainly deal with management issues and only some farm stakeholders are involved in them. But all stakeholders need to have a voice and be aware of the biosecurity problems on farms, and all levels of the government services need to take these problems into consideration. Moreover, the agreements that could be reached at these meetings could be binding, as long as they do not affect or are not affected by existing legislation.

Biosecurity meetings were not fully discussed and clarified in terms of who attends them and how, or what their aims were, a situation that should be further discussed among all farm stakeholders in view of their respective responsibilities. Furthermore, it is important to consider that the responsibility for routine biosecurity does not only lie with farmers, but also with other actors, including the dairy industry and transport companies, as well as the government services (65). In this regard, biosecurity meetings could be designed on the basis of participatory methods with consensus-based decisions across the board (66). Similar initiatives could be considered for this design, such as those carried out by Bugeza et al. (67) and Vaarst et al. (68), or those evidenced through AgriLink (69) and LIVERUR (70). These meetings and their decisions could generate greater awareness and commitment among their participants, as already occurs, for instance, in healthcare with patients, thus generating realistic and informed expectations about healthcare and increasing satisfaction and trust in it (71, 72); or in corporate organizations with employees, who are able to contribute to the organisation's productivity (73).

It is important to note that there are practically no studies involving government veterinary services on the issue of biosecurity (24, 25). Hence this is the first contribution to offer evidence from the perspective of government veterinarians through their discourses on biosecurity on livestock farms, and specifically dairy cattle farms in Galicia and Catalonia. Government veterinarians may share the opinions of farmers and private veterinarians on the intervention of government veterinary services in the implementation of biosecurity measures, but they may be constrained by the regulations that they have to enforce. Despite this, biosecurity regulations could be viewed as an advantage for government veterinary services, as they are generally absent on dairy cattle farms, only a few of which are linked to TB and IBR. This would increase the scope for dialogue between government veterinary services and farmers and private veterinarians to agree on and adjust biosecurity measures in accordance with the real context of the affected farms.

Government veterinarians are aware that the services they represent prioritize a sanctioning rather than an advisory role. This situation can be a drawback for the implementation of biosecurity. Therefore, the interventions carried out by government veterinary services should consider and reinforce their interaction with other actors in order to gain a better understanding of the reality of the farms and to adapt their advisory role to these contexts, and thus support the implementation of biosecurity measures. In addition, these interventions could also assist the transition toward the implementation of mandatory biosecurity measures that could be enacted in future regulations.

Therefore, it could be interesting to incorporate government veterinary services in future studies on biosecurity or related topics, as there is a tendency for farmers and veterinarians to question them, and this would give them the chance to share their own perspective. Better knowledge of all stakeholders' perspectives would also mean a better understanding of the psychosocial dynamics involved in such matters as heterogeneity in the routine implementation of biosecurity measures (9, 74–76). Furthermore, from a health perspective, stakeholders could also include public health professionals, especially in cases of zoonotic infectious diseases.

In conclusion, government veterinary services have a similar perspective to that of farmers and veterinarians. They generally disagree with actions and initiatives that prioritize sanctions over advice, and claim to be willing to visit farms to learn about farmers' realities and their problems with routine biosecurity, thus leading to greater flexibility of certain regulations that may be complicated for farmers to carry out. Government veterinary services do try to balance their own institutional perspectives with those of the sector, such as those of farmers and veterinarians, to ensure that biosecurity measures are implemented efficiently and effectively on a day-to-day basis. Thus, this study is useful for the generation and improvement of biosecurity interventions and regulations involving government veterinarians, which will help to regain farmers' trust in the government services by reinforcing their interaction with other stakeholders and their advisory role in the implementation of biosecurity measures. Hence, the government veterinary services and the dairy cattle sector will benefit from this study. Government veterinary services could intervene internally at their different levels to improve the performance of their government veterinarians in the institution and in the field, while the dairy sector could improve the implementation of routine biosecurity measures. Therefore, it is relevant to consider the perceptions of government veterinary services in biosecurity studies. This in turn makes it possible to appreciate the small differences within the different levels of the same institution.

## 5. Limitations

Eleven interviews were conducted with government veterinarians belonging to the four territorial levels of the government veterinary services in two ACs. However, the population sample is only representative of Galicia and Catalonia and not of all 17 ACs in Spain. Government veterinarians belonging to the other ACs may have different opinions from those in Galicia and Catalonia, even though the dairy cattle farms present in other ACs may be similar to those of Galicia and Catalonia. These results are framed within a particular context with its own legislations and regulations, and it is hard to compare them with other results obtained with the same methodology, both in other regions of Spain and in different countries.

Also, only government veterinarians with knowledge and experience of livestock farm biosecurity were considered. Government veterinarians could have knowledge and experience not only in dairy cattle farming, but also in other production systems, such as pig and poultry farming. The methodology could therefore be replicated in other farming sectors. The results related to these livestock production systems and their regulations could be different from those of this study, but the results linked to the government services, roles of government veterinarians and participatory processes could be similar. This is because dairy cattle farms are managed differently from pig and poultry farms, which tend to have higher levels of biosecurity due to the stricter mandatory regulations on the latter. For example, pig farms are governed by Royal Decree 324/2000 (77), Royal Decree 3483/2000 (78), and Royal Decree 1221/2009 (79) mainly related to management, and poultry farms are affected by Order APA/2442/2006 (80) and Royal Decree 445/2007 (81) mainly related to avian influenza. There are no regulations on mandatory measures for dairy cattle farms other than the stipulations of Regulation 2016/429 (26). These more stringent measures for pig and poultry farms have mainly come about as a result of recent outbreaks of African swine fever and avian influenza (82, 83).

## Data availability statement

The raw data supporting the conclusions of this article will be made available by the authors, without undue reservation.

## Ethics statement

The studies involving human participants were reviewed and approved by the Ethics Committee of the Universitat Autònoma de Barcelona (CEEAH 4055). The patients/participants provided their written informed consent to participate in this study.

## Author contributions

SM conducted the study, wrote the article as part of his doctoral thesis (2017–2021), collected, analyzed, and presented the data, along with its critical aspects. AA was SM's supervisor and was extensively involved throughout the study and draft of this article. JC and AA recruited government veterinarians at the national and autonomous community levels in Catalonia. EY and FD recruited government veterinarians at the autonomous community, provincial and county levels in Galicia. All authors approved the article submitted for publication.

## References

[1] World Organisation for Animal Health. Biosecurity. Glossary (2022). Available online at: https://www.woah.org/fileadmin/Home/eng/Health_standards/tahc/current/glossaire.pdf (Accessed August 28, 2022).

[2] European Commission. How Are Farmers Dealing With Biosecurity? Healthy Animals, Less Medication Better Prices Through Biosecurity. (2016). Available online at: https://ec.europa.eu/eip/agriculture/sites/agri-eip/files/2015-press11-biosecurity_final.pdf (Accessed August 28, 2022).

[3] Frössling J Nöremark M. Differing perceptions: Swedish farmers' views of infectious disease control. Vet Med Sci. (2016) 2:54–68. 10.1002/vms3.20 29067181 PMC5645822

[4] Laanen M Maes D Hendriksen C Gelaude P De Vliegher S Rosseel Y. Pig, cattle and poultry farmers with a known interest in research have comparable perspectives on disease prevention and on-farm biosecurity. Prev Vet Med. (2014) 115:1–9. 10.1016/j.prevetmed.2014.03.015 24703250

[5] Toma L Stott A Heffernan C Ringrose S Gunn G. Determinants of biosecurity behaviour of British cattle and sheep farmers-a behavioural economics analysis. Prev Vet Med. (2013) 108:321–33. 10.1016/j.prevetmed.2012.11.009 23194894

[6] Kuster K Cousin M Jemmi T Schüpbach-Regula G Magouras I. Expert opinion on the perceived effectiveness and importance of on-farm biosecurity measures for cattle and swine farms in Switzerland. PLoS ONE. (2015) 10:e0144533. 10.1371/journal.pone.0144533 26656893 PMC4686079

[7] Toma L Low J Vosough B Matthews L Stott A. An analysis of cattle farmers' perceptions of drivers and barriers to on-farm control of *Escherichia coli* O157. Epidemiol Infect. (2015) 143:2355–66. 10.1017/S0950268814003045 25427776 PMC9151035

[8] García J Coelho A. Evaluación del conocimiento de los ganaderos sobre la tuberculosis bovina e implicaciones para su control. Rev Mex Cienc Pecu. (2014) 5:213–29. 10.22319/rmcp.v5i2.3662

[9] Brennan M Christley R. Biosecurity on cattle farms: a study in north-west England. PLoS ONE. (2012) 7:1–8. 10.1371/journal.pone.0028139 22235244 PMC3250388

[10] Broughan J Maye D Carmody P Brunton L Ashton A Wint W. Farm characteristics and farmer perceptions associated with bovine tuberculosis incidents in areas of emerging endemic spread. Prev Vet Med. (2016) 129:88–98. 10.1016/j.prevetmed.2016.05.007 27317326

[11] Renault V Humblet M Moons V Bosquet G Gauthier B Cebrián L. Rural veterinarian's perception and practices in terms of biosecurity across three European countries. Transbound Emerg Dis. (2018) 65:e183–93. 10.1111/tbed.12719 28940807

[12] Ciaravino G Ibarra P Casal E López S Espluga J Casal J Napp S Allepuz A. Farmer and veterinarian attitudes towards the bovine tuberculosis eradication programme in Spain: What is going on in the field? Front Vet Sci. (2017) 4:202. 10.3389/fvets.2017.00202 29230403 PMC5712013

[13] Sayers R Good M Sayers G A. survey of biosecurity-related practices, opinions and communications across dairy farm veterinarians and advisors. Vet J. (2014) 200:261–9. 10.1016/j.tvjl.2014.02.010 24679454

[14] Heffernan C Nielsen L Thomson K Gunn G. An exploration of the drivers to biosecurity collective action among a sample of UK cattle and sheep farmers. Prev Vet Med. (2008) 87:358–72. 10.1016/j.prevetmed.2008.05.007 18692923

[15] Cardwell J Van Winden S Beauvais W Mastin A De Glanville W Hardstaff J. Assessing the impact of tailored biosecurity advice on farmer behaviour and pathogen presence in beef herds in England and Wales. Prev Vet Med. (2016) 135:9–16. 10.1016/j.prevetmed.2016.10.018 27931934

[16] Shortall O Ruston A Green M Brennan M Wapenaar W Kaler J. Broken biosecurity? Veterinarians' framing of biosecurity on dairy farms in England. Prev Vet Med. (2016) 132:20–31. 10.1016/j.prevetmed.2016.06.001 27664445

[17] Ellis-Iversen J Cook A Watson E Nielen M Larkin L Wooldridge M. Perceptions, circumstances and motivators that influence implementation of zoonotic control programs on cattle farms. Prev Vet Med. (2010) 93:276–85. 10.1016/j.prevetmed.2009.11.005 19963291

[18] Moya S Tirado F Espluga J Ciaravino G Armengol R Diéguez J. Dairy farmers' decision making to implement biosecurity measures: a study of psychosocial factors. Transbound Emerg Dis. (2019) 67:698–710. 10.1111/tbed.13387 31600857 PMC7079090

[19] Moya S Tirado F Diéguez F Allepuz A. From biosecurity to security ecologies: an analysis between old dairy farming traditions and routines and veterinary recommendations in Spain. Sociol Ruralis. (2020) 61:372–97. 10.1111/soru.12333

[20] Sayers R Sayers G Mee J Good M Bermingham M Grant J. Implementing biosecurity measures on dairy farms in Ireland. Vet J. (2013) 197:259–67. 10.1016/j.tvjl.2012.11.017 23276712

[21] Hoe F Ruegg P. Opinions and practices of Wisconsin dairy producers about biosecurity and animal well-Being. J Dairy Sci. (2006) 89:2297–308. 10.3168/jds.S0022-0302(06)72301-3 16702297

[22] Pritchard K Wapenaar W Brennan M. Cattle veterinarians' awareness and understanding of biosecurity. Vet Rec. (2015) 176:546–8. 10.1136/vr.102899 25854278 PMC4453591

[23] Gunn G Heffernan C Hall M McLeod A Hovi M. Measuring and comparing constraints to improved biosecurity amongst GB farmers, veterinarians and the auxiliary industries. Prev Vet Med. (2008) 84:310–23. 10.1016/j.prevetmed.2007.12.003 18282623

[24] Kristensen E Jakobsen E. Danish dairy farmers' perception of biosecurity. Prev Vet Med. (2011) 99:122–9. 10.1016/j.prevetmed.2011.01.010 21345504

[25] Hovi M Mcleod A Gunn G. Assessing UK farmer attitudes to biosecurity on sheep and cattle farms. Res Vet Sci. (2005) 78:1–46.

[26] Regulation. Official Journal of the European Union. Strasbourg: Official Journal of the European Union (2016).

[27] Ministerio de Agricultura, Pesca y, Alimentación,. Consulta Pública del Proyecto de Real Decreto por el que se Establece la Normativa Básica de Ordenación de las Granjas de ganado bovino. (2019). Available online at: https://www.mapa.gob.es/es/ganaderia/participacion-publica/consultapreviardordenacionbovina_tcm30-502616.pdf (Accessed August 28, 2022).

[28] Denis-Robichaud J Kelton D Bauman C Barkema H Keefe G Dubuc J. Canadian dairy farmers' perception of the efficacy of biosecurity practices. J Dairy Sci. (2019) 102:10657–69. 10.3168/jds.2019-16312 31477301

[29] Damiaans B Sarrazin S Heremans E Dewulf J. Perception, motivators and obstacles of biosecurity in cattle production. Vlaams Diergeneeskd Tijdschr. (2018) 87:150–63. 10.21825/vdt.v87i3.16079

[30] Shortall O Green M Brennan M Wapenaar W Kaler J. Exploring expert opinion on the practicality and effectiveness of biosecurity measures on dairy farms in the United Kingdom using choice modeling. J Dairy Sci. (2017) 100:2225–39. 10.3168/jds.2016-11435 28088420

[31] Brennan M Wright N Wapenaar W Jarratt S Hobson-West P Richens I. Exploring attitudes and beliefs towards implementing cattle disease prevention and control measures: a qualitative study with dairy farmers in Great Britain. Animals. (2016) 6:61. 10.3390/ani6100061 27727168 PMC5082307

[32] Moya S Chan K Hinchliffe S Buller H Espluga J Benavides B. Influence on the implementation of biosecurity measures in dairy cattle farms: Communication between veterinarians and dairy farmers. Prev Vet Med. (2021) 190:105329. 10.1016/j.prevetmed.2021.105329 33756432

[33] Christley R Robinson S Moore B Setzkorn C Donald I. Responses of farmers to introduction in England and Wales of pre-movement testing for bovine tuberculosis. Prev Vet Med. (2011) 100:126–33. 10.1016/j.prevetmed.2011.02.005 21377746

[34] Enticott G. The ecological paradox: social and natural consequences of the geographies of animal health promotion. Trans Inst Br Geogr. (2008) 33:433–46. 10.1111/j.1475-5661.2008.00321.x

[35] Renault V Humblet M Pham P Saegerman C. Biosecurity at cattle farms: Strengths, weaknesses, opportunities and threats. Pathogens. (2021) 10:1315. 10.3390/pathogens10101315 34684268 PMC8538770

[36] Kogan M. Researching the powerful in education and elsewhere. In: Walford G, editor. Researching the Powerful in Education. London: UCL Press (1994). p. 67–80.

[37] Kennedy A Christie D Fraser C Reid L McKinney S Welsh M. Key informants' perspectives on teacher learning in Scotland. Br J Educ Stud. (2008) 56:400–19. 10.1111/j.1467-8527.2008.00416.x

[38] Marshall M. The key informant technique. Fam Pract. (1996) 13:92–7. 10.1093/fampra/13.1.92 8671109

[39] Pillai S Siddika N Apu E Kabir R. COVID-19: Situation of European countries so far. Arch Med Res. (2020) 51:723–5. 10.1016/j.arcmed.2020.05.015 32475614 PMC7242919

[40] Henríquez J Gonzalo-Almorox E García-Goñi M Paolucci F. The first months of the COVID-19 pandemic in Spain. Health Policy Technol. (2020) 9:560–74. 10.1016/j.hlpt.2020.08.013 32874852 PMC7451011

[41] Jones R Abdelfattah K. Virtual interviews in the era of COVID-19: a primer for applicants. J Surg Educ. (2020) 77:733–4. 10.1016/j.jsurg.2020.03.020 32278546 PMC7142702

[42] Joshi A Bloom D Spencer A Gaetke-Udager K Cohan R. Video interviewing: a review and recommendations for implementation in the era of COVID-19 and beyond. Acad Radiol. (2020) 27:1316–22. 10.1016/j.acra.2020.05.020 32563558 PMC7833741

[43] Davis M Haas M Gottlieb M House J Huang R Hopson L. Zooming in versus flying out: virtual residency interviews in the era of COVID-19. AEM Educ Train. (2020) 4:443–6. 10.1002/aet2.10486 33150292 PMC7592818

[44] Longhurst R. Interviews: In-depth, semi-structured. In: Kitchin R Thrift N, editors. International Encyclopedia of Human Geography. Amsterdam: Elsevier (2009). p. 580–4. 10.1016/B978-008044910-4.00458-2

[45] Elo S Kyngas H. The qualitative content analysis process. J Adv Nurs. (2008) 62:107–15. 10.1111/j.1365-2648.2007.04569.x 18352969

[46] Elo S Kaariainen M Kanste O Polkki T Utriainen K Kyngas H. Qualitative content analysis: a focus on trustworthiness. SAGE Open. (2014) 4:1–10. 10.1177/2158244014522633

[47] Hsieh H Shannon S. Three approaches to qualitative content analysis. Qual Health Res. (2005) 15:1277–88. 10.1177/1049732305276687 16204405

[48] Creswell J Poth C. Qualitative Inquiry and Research Design: Choosing Among Five Approaches. Los Angeles: SAGE Publications (2013).

[49] Morse J Barrett M Mayan M Olson K Spiers J. Verification strategies for establishing reliability and validity in qualitative research. Int J Qual Methods. (2002) 1:13–22. 10.1177/16094069020010020218626033

[50] Hennink M Kaiser B. Sample sizes for saturation in qualitative research: a systematic review of empirical tests. Soc Sci Med. (2022) 292:114523. 10.1016/j.socscimed.2021.114523 34785096

[51] Ministerio de Agricultura, Pesca y, Alimentación,. Programa Nacional de Erradicación de Tuberculosis Bovina Presentado por España para el año 2020. (2020). Available online at: https://www.mapa.gob.es/es/ganaderia/temas/sanidad-animal-higiene-ganadera/pnetb_2020final_tcm30-523317.PDF (Accessed August 28, 2022).

[52] Royal Decree 2611/1996. Agencia Estatal Boletín Oficial del Estado. Madrid, Spain (1996).

[53] Royal Decree 554/2019. Agencia Estatal Boletín Oficial del Estado. Madrid, Spain (2019).

[54] Oliveira V Anneberg I Voss H Sørensen J Thomsen P. Attitudes of danish dairy farmers towards biosecurity. Livest Sci. (2018) 214:153–60. 10.1016/j.livsci.2018.06.004

[55] McWilliam W Balzarova M. The role of dairy company policies in support of farm green infrastructure in the absence of government stewardship payments. Land Use Policy. (2017) 68:671–80. 10.1016/j.landusepol.2017.08.030

[56] Ministerio de Agricultura, Pesca y, Alimentación,. Imágenes de un Mundo Rural 1955-1980. (2006). Available online at: https://www.mapa.gob.es/es/ministerio/archivos-bibliotecas-mediateca/mediateca/imagenes_mundo_rural_tcm30-90020.pdf (Accessed August 28, 2022).

[57] Royal Decree 993/2014. Agencia Estatal Boletín Oficial del Estado. Madrid, Spain (2014).

[58] Law 8/2003. Agencia Estatal Boletín Oficial del Estado. Madrid, Spain (2006).

[59] Derks M Van Werven T Hogeveen H Kremer W. Veterinary herd health management programs on dairy farms in the Netherlands: use, execution, and relations to farmer characteristics. J Dairy Sci. (2013) 96:1623–37. 10.3168/jds.2012-6106 23357015

[60] Derks M Van de Ven L Van Werven T Kremer W Hogeveen H. The perception of veterinary herd health management by Dutch dairy farmers and its current status in the Netherlands: a survey. Prev Vet Med. (2012) 104:207–15. 10.1016/j.prevetmed.2011.12.019 22284342

[61] Da Silva J Noordhuizen J Vagneur M Bexiga R Gelfert C Baumgartner W. Veterinary dairy herd health management in Europe: constraints and perspectives. Vet Q. (2006) 28:23–32. 10.1080/01652176.2006.9695203 16605158

[62] Lievaart J Noordhuizen J. Veterinary herd health management on dairy farms in the Netherlands: Assessment by dairy farmers. Tijdschr Diergeneeskd. (1999) 124:734–40. 10635105

[63] Paquette C Schemann K Ward M. Knowledge and attitudes of Australian livestock producers concerning biosecurity practices. Aust Vet J. (2020) 98:533–45. 10.1111/avj.13005 32754999

[64] Moore D Merryman M Hartman M Klingborg D. Comparison of published recommendations regarding biosecurity practices for various production animal species and classes. J Am Vet Med Assoc. (2008) 233:249–56. 10.2460/javma.233.2.249 18627227

[65] Maye D Chan K. On-farm biosecurity in livestock production: farmer behaviour, cultural identities, and practices of care. Emerg Top Life Sci. (2020) 4:521–30. 10.1042/ETLS20200063 32909609

[66] Cioni L. Participative Methods Consensus Theory (Technical Report: TR-08-23). (2008). Available online at: https://citeseerx.ist.psu.edu/viewdoc/downloadjsessionid=D0778437B61B6015A13B97EA911FDCF3?doi=10.1.1.163.9862&rep=rep1&type=pdf (Accessed August 28, 2022).

[67] Bugeza J Kankya C Muleme J Akandinda A Sserugga J Nantima N. Participatory evaluation of delivery of animal health care services by community animal health workers in Karamoja region of Uganda. PLoS ONE. (2017) 12:e0179110. 10.1371/journal.pone.0179110 28594945 PMC5464622

[68] Vaarst M Byarugaba D Nakavuma J Laker C. Participatory livestock farmer training for improvement of animal health in rural and peri-urban smallholder dairy herds in Jinja, Uganda. Trop Anim Health Prod. (2007) 39:1–11. 10.1007/s11250-006-4439-8 17941482

[69] AgriLink. Living Labs. (2019). Available online at: https://old.agrilink2020.eu/our-work/living-labs/ (Accessed August 28, 2022).

[70] European Commission. Living Lab Research Concept in Rural Areas. (2018). Available online at: https://cordis.europa.eu/project/id/773757/es (Accessed August 28, 2022).

[71] Vahdat S Hamzehgardeshi L Hessam S Hamzehgardeshi Z. Patient involvement in health care decision making: a review. Iran Red Crescent Med J. (2014) 16:12454. 10.5812/ircmj.12454 24719703 PMC3964421

[72] WHO. Exploring Patient Participation in Reducing Health-Care-Related Safety Risks. (2013). Available online at: https://www.euro.who.int/__data/assets/pdf_file/0010/185779/e96814.pdf (Accessed October 14, 2022).

[73] Elegbe O Ibikunle F. Effective communication and participative decision-making in selected organizations in Ibadan metropolis. Afr J Stab Dev. (2015) 9:38–54.

[74] Denis-Robichaud J Kelton D Bauman C Barkema H Keefe G Dubuc J. Biosecurity and herd health management practices on Canadian dairy farms. J Dairy Sci. (2019) 102:9536–47. 10.3168/jds.2018-15921 31351735

[75] Renault V Damiaans B Sarrazin S Humblet M Dewulf J Saegerman C. Biosecurity practices in Belgian cattle farming: level of implementation, constraints and weaknesses. Transbound Emerg Dis. (2018) 65:1246–61. 10.1111/tbed.12865 29566303

[76] Sahlström L Virtanen T Kyyrö J Lyytikäinen T. Biosecurity on Finnish cattle, pig and sheep farms - Results from a questionnaire. Prev Vet Med. (2014) 117:59–67. 10.1016/j.prevetmed.2014.07.004 25147126

[77] Royal Decree 324/2000. Agencia Estatal Boletín Oficial del Estado. Madrid, Spain, (2000).

[78] Royal Decree 3483/2000. Agencia Estatal Boletín Oficial del Estado. Madrid, Spain (2000).

[79] Royal Decree 1221/2009. Agencia Estatal Boletín Oficial del Estado. Madrid, Spain (2009).

[80] Order APA/2442/2006. A*gencia Estatal Bolet*í*n Oficial del Estado*. Madrid, Spain (2006).

[81] Royal Decree 445/2007. Agencia Estatal Boletín Oficial del Estado. Madrid, Spain (2007).

[82] World Organisation for Animal Health. African Swine Fever (ASF) – Situation Report 20. (2022). Available online at: https://www.woah.org/app/uploads/2022/09/asf-report20.pdf (Accessed October 17, 2022).

[83] European Centre for Disease Prevention Control. Avian Influenza Overview June – September 2022. (2022). Available online at: https://www.ecdc.europa.eu/sites/default/files/documents/avian-influenza-overview-September-2022_0.pdf (Accessed October 17, 2022).

